# Medical image segmentation based on self-supervised hybrid fusion network

**DOI:** 10.3389/fonc.2023.1109786

**Published:** 2023-04-14

**Authors:** Liang Zhao, Chaoran Jia, Jiajun Ma, Yu Shao, Zhuo Liu, Hong Yuan

**Affiliations:** ^1^ School of Software Technology, Dalian University of Technology, Dalian, China; ^2^ The First Affiliated Hospital of Dalian Medical University, Dalian, China; ^3^ The Affiliated Central Hospital, Dalian University of Technology, Dalian, China

**Keywords:** self-supervised learning, multi-modal, hybrid fusion, medical image segmentation, medical image segmentation based on self-supervised hybrid fusion network

## Abstract

Automatic segmentation of medical images has been a hot research topic in the field of deep learning in recent years, and achieving accurate segmentation of medical images is conducive to breakthroughs in disease diagnosis, monitoring, and treatment. In medicine, MRI imaging technology is often used to image brain tumors, and further judgment of the tumor area needs to be combined with expert analysis. If the diagnosis can be carried out by computer-aided methods, the efficiency and accuracy will be effectively improved. Therefore, this paper completes the task of brain tumor segmentation by building a self-supervised deep learning network. Specifically, it designs a multi-modal encoder-decoder network based on the extension of the residual network. Aiming at the problem of multi-modal feature extraction, the network introduces a multi-modal hybrid fusion module to fully extract the unique features of each modality and reduce the complexity of the whole framework. In addition, to better learn multi-modal complementary features and improve the robustness of the model, a pretext task to complete the masked area is set, to realize the self-supervised learning of the network. Thus, it can effectively improve the encoder’s ability to extract multi-modal features and enhance the noise immunity. Experimental results present that our method is superior to the compared methods on the tested datasets.

## 1 Introduction

In recent years, medical image segmentation has become a hot topic in the area of medicine, and its purpose is to clearly show the changes of anatomical or pathological structures in medical images. Popular medical image segmentation tasks include liver, brain and its tumor segmentation, optic disc segmentation and cell segmentation, lung and lung nodule segmentation, etc. Brain tumor segmentation is considered to be one of the most challenging problems in this field (1). And computer-aided segmentation of medical images has become a highly valuable research content (2). In order to help clinicians make accurate judgments, it is necessary to extract and segment some key targets of medical images (3, 4).

Therefore, many research works have proposed different models for the medical image segmentation problem. For example, encoder-decoder based segmentation models are widely used in medical image segmentation (5). In order to solve the medical image segmentation problem, Ronneberger et al. (6) proposed the U-Net model, which won several firsts in the cell-level segmentation task competition at that time. Due to the characteristics of medical imaging devices, multi-modal is often involved in medical applications (7). In recent years, although the network models applied to medical image processing are mainly focused on single-modal models, there are still studies on multi-modal network models, which makes up for the shortcomings of single-modal models in dealing with different modalities (8, 9). However, medical images are difficult to obtain, resulting in the small amount of data (10). This problem is more pronounced in multi-modal analysis, because such learning methods require more modalities of data and are more demanding on the dataset. In addition, due to the noise in medical images and the subtle differences between human organs, the automatic segmentation of medical images also requires strong robustness of the network. Besides, most of the current medical image segmentation research work is supervised learning, which often requires a large amount of data. Therefore, the self-supervised learning method is more advantageous in this case. It can achieve the training effect with less data, which is especially suitable for multi-modal networks (11).

Medical image segmentation relies heavily on large labeled datasets, which are difficult to achieve due to the expense and time required to generate expert annotations. Self-supervised learning offers a promising solution to this problem by using unsupervised pre-training on unlabeled data, which can reduce the burden of manual annotation. However, most self-supervised learning approaches neglect the multi-modal nature of medical images, which is essential for accurate analysis and diagnosis, and integrating cross-modal information is necessary for effective segmentation. Chaitanya et al. (12) proposed a semi-supervised approach to volumetric medical image segmentation that extends the contrastive learning framework by leveraging domain-specific and problem-specific cues, and achieved substantial improvements over other self-supervision and semi-supervised learning techniques. Wu et al. (13) proposed a federated contrastive learning framework for volumetric medical image segmentation with limited annotations, which exchanged features in the pre-training process to provide diverse contrastive data to each site for effective local contrastive learning while keeping raw data private. Taleb et al. (14) proposed a self-supervised method that leverages multiple imaging modalities through a multimodal puzzle task, which confused image modalities at the data-level to learn a modality-agnostic feature embedding, and utilized cross-modal generation techniques for multimodal data augmentation, achieving better semantic representations and improved data-efficiency. Taleb et al. (15) proposed 3D versions of five different self-supervised methods in the form of proxy tasks, facilitating neural network feature learning from unlabeled 3D images, and yielding more powerful semantic representations that enable solving downstream tasks more accurately and efficiently, even when transferring the learned representations to a small downstream-specific dataset. Zou et al. (16) presented a trusted brain tumor segmentation network that generates robust segmentation results and reliable uncertainty estimations. The model uncertainty used subjective logic theory and gathers reliable evidence from the features to obtain the final segmentation results. The proposed model is evaluated on the BraTS, 2019 (17) dataset through qualitative and quantitative experiments. Li et al. (18) proposed Segtran, an alternative segmentation framework based on transformers for medical image segmentation. Segtran incorporated large context and high spatial resolutions, resulting in unlimited “effective receptive fields” even at high feature resolutions.

Inspired by these, we propose a self-supervised multi-modal brain tumor segmentation framework with hybrid fusion of modality-specific features (SM-ResUNet). We innovatively extend Res-UNet (19, 20) into a multi-modal network, introduce the modal fusion and attention methods in each skip connection, and implement a self-supervised learning mechanism for improving network robustness and optimizing its performance. We design a pretext task capable of exploiting cross-modal information rather than simply using single image information as in most previous studies. Multi-modal networks are able to use unique encoders for feature extraction for each modality, and the multi-modal network structure can be complementary to the assisted task design model, so multi-modal is necessary. Moreover, we design a novel feature fusion scheme to support input of different numbers of modalities, and capture the relevant information of each modality feature through an attention mechanism. On this basis, the multi-modal Res-UNet is employed as the backbone structure of the model, which is suitable for the segmentation task of medical images. The SM-ResUNet is based on a semantic segmentation architecture of multi-modal input, which makes full use of independent features in multi-modal data. During the training process, the overfitting problem caused by small datasets can be alleviated by jointly training a pretext task with the segmentation network. We validate the effectiveness of the SM-ResUNet on the BraTS brain tumor segmentation dataset. Experiments show that the SM-ResUNet is overall better than other compared models, which proves the effectiveness and usability of the SM-ResUNet.

## 2 Method

**Figure 1:**
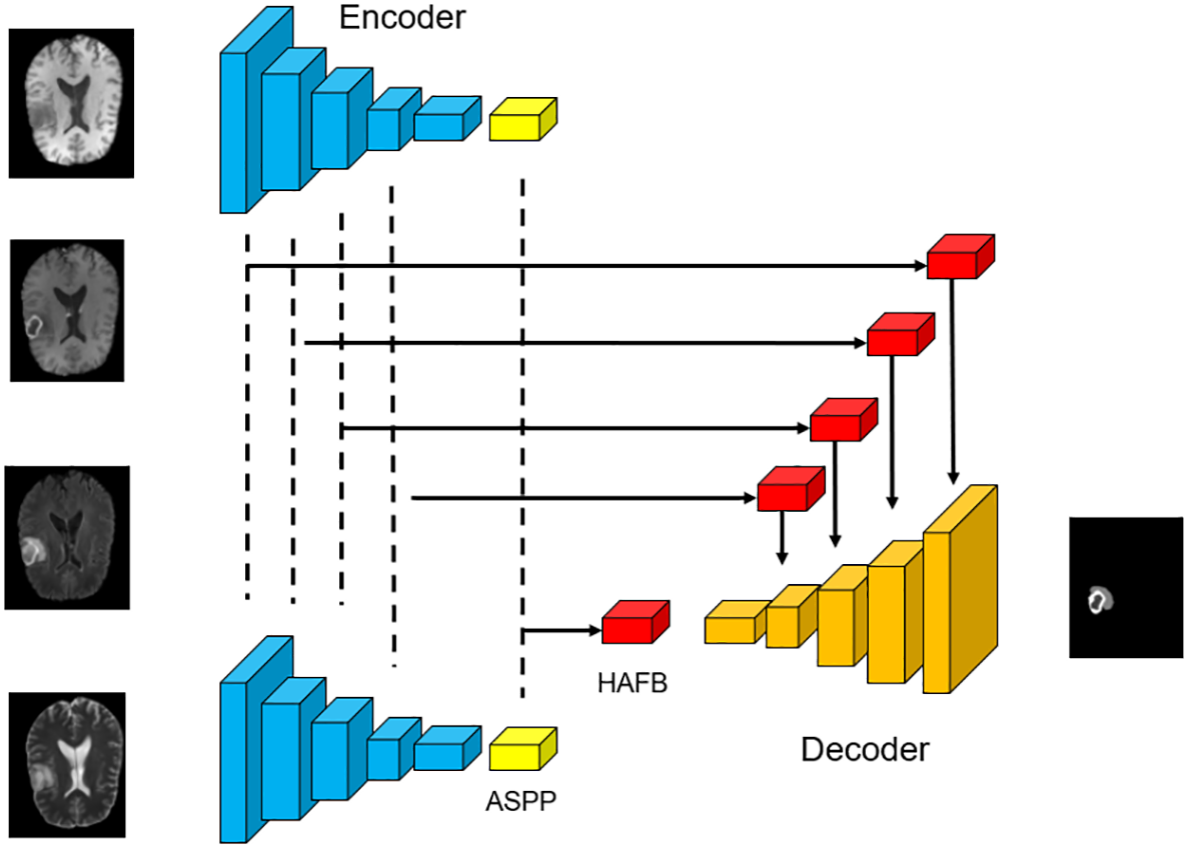
The overall structure of the network. The basic framework used in this work is Res-UNet with skip connections and residual convolution, which is suitable for automatic segmentation of brain tumor medical images.

The SM-ResUNet we designed is shown in **Figure 1**, the network is implemented based on Res-UNet, and a multi-modal mechanism is introduced. The multi-modal features are fused through Hybrid Attentional Fusion Block (HAFB), and the attention mechanism is employed to extract valuable information (21). At the bottleneck layer between the encoder and the decoder, each multi-modal feature is extracted with different receptive fields through Atrous Spatial Convolutional Pyramid Structure (ASPP) (22–24), so as to make full use of the valuable information in each modality. In addition, the self-supervised learning is introduced into the network to improve the robustness of the network and the feature extraction ability of the encoder. Specifically, in Section 1, we will describe the multi-modal encoder and decoder structure and its function in our proposed network. In Section 2, we will introduce the HAFB module in detail. In Section 3, we will present the implementation of the self-supervised learning mechanism and illustrate the effectiveness of this approach.

### 2.1 Multi-modal encoder and decoder

To support multi-modal inputs, multiple encoders are used in the network to achieve feature extraction for each modality. The network structure includes a decoder, which restores the fused multi-modal features to the original image size through several residual convolutions and upsampling, and obtains the segmentation result.

Four encoders are adopted in the network, and each encoder is used to perform feature extraction on its corresponding modality to obtain independent latent features. Each encoder has four layers of the same structure, which contains one residual convolution and downsampling. After residual convolution, the network obtains a feature map with the same size as the input image and different number of channels. Then the feature map goes through the pooling layer, which changes the image size to 1/2 of the original size.

After repeating the above process four times, each encoder outputs a feature map that is 1/16 the size of the original image. Each encoder is calculated as follows, 


(1) $$Conv\;(x)=ReLU\;\left(BN\left(\varphi_{3\times 3}(x)\right)\right)$$

(2) $$ResConv\;(x)=\varphi_{1\times 1}(x)+Conv_{2}\left(DP\left(Conv_{1}(x)\right)\right)$$

(3) EL (x)=MP(ResConv(x))

(4) $$Encode\;(x)=EL_{4}\left(EL_{3}\left(EL_{2}\left(EL_{1}(x)\right)\right)\right)$$

where $\varphi_{n\times n}$ represents the convolution operation with a convolution kernel size of *n*×*n*, *BN* is the batchnorm layer, *DP* is the dropout layer, and *MP* is the max pooling layer. $EL_{i}$ represents the layer *i* of the encoder. The number of channels will not be changed by $Conv_{2}$ , except that the first layer is determined according to the number of initial convolution kernels. The number of output feature map channels of $Conv_{1}$ and $\varphi_{1\times 1}$ are twice the number of the input in *ResConv*.

Each decoder in the network ends up with a specific ASPP structure (23). The ASPP employs multiple dilation rate convolution kernels to obtain latent features, and can sample the given input through dilated convolution at different sampling rates to capture the context of the feature map at multiple scales, so as to more accurately locate different size of brain tumor. The input to each ASPP is the feature map of its corresponding modality.

Corresponding to the encoder, a decoder needs to be introduced into the network structure to decode the output of the encoder. It contains multiple upsampling, to restore the feature map to the original image size, and to determine the segmentation result of each local feature. In the last layer of the decoder, a convolution is adopted to change the number of channels into the number of final segmentation types. Each channel corresponds to a classification, and the value of the pixel at the corresponding position represents the score in that classification. After four times of upsampling and residual convolution, the decoder will output a feature map that is 16 times the size of the output of the encoder. For the decoder, the following operations are performed,


(5) DL (x)=ResConv(TransConv(x))

(6) $$Decode\;(x)=DL_{1}\left(DL_{2}\left(DL_{3}\left(DL_{4}(x)\right)\right)\right)$$

where *ResConv* is calculated in the same way as Equation 1-2. *TransConv* is transpose convolution. $DL_{i}$ represents the layer *i* of the decoder. At the end of the decoder, a convolution of size 3×3 is set up, so that the number of image channels is the same as the number of segmentation types.

### 2.2 Hybrid attentional fusion block in skip connection

We employ the soft attention mechanism, HAFB (25), for multi-modal fusion and place it in skip connections in the network. In each skip connection of the network, HAFB is integrated for multi-modal fusion of the features in the same layer of encoder. This structure combines a multi-modal fusion method on the basis of an attention structure, which is suitable for the multi-modal network structure used in this paper, and can retain the representative features of each modality while maintaining the stability of the network.

**Figure 2:**
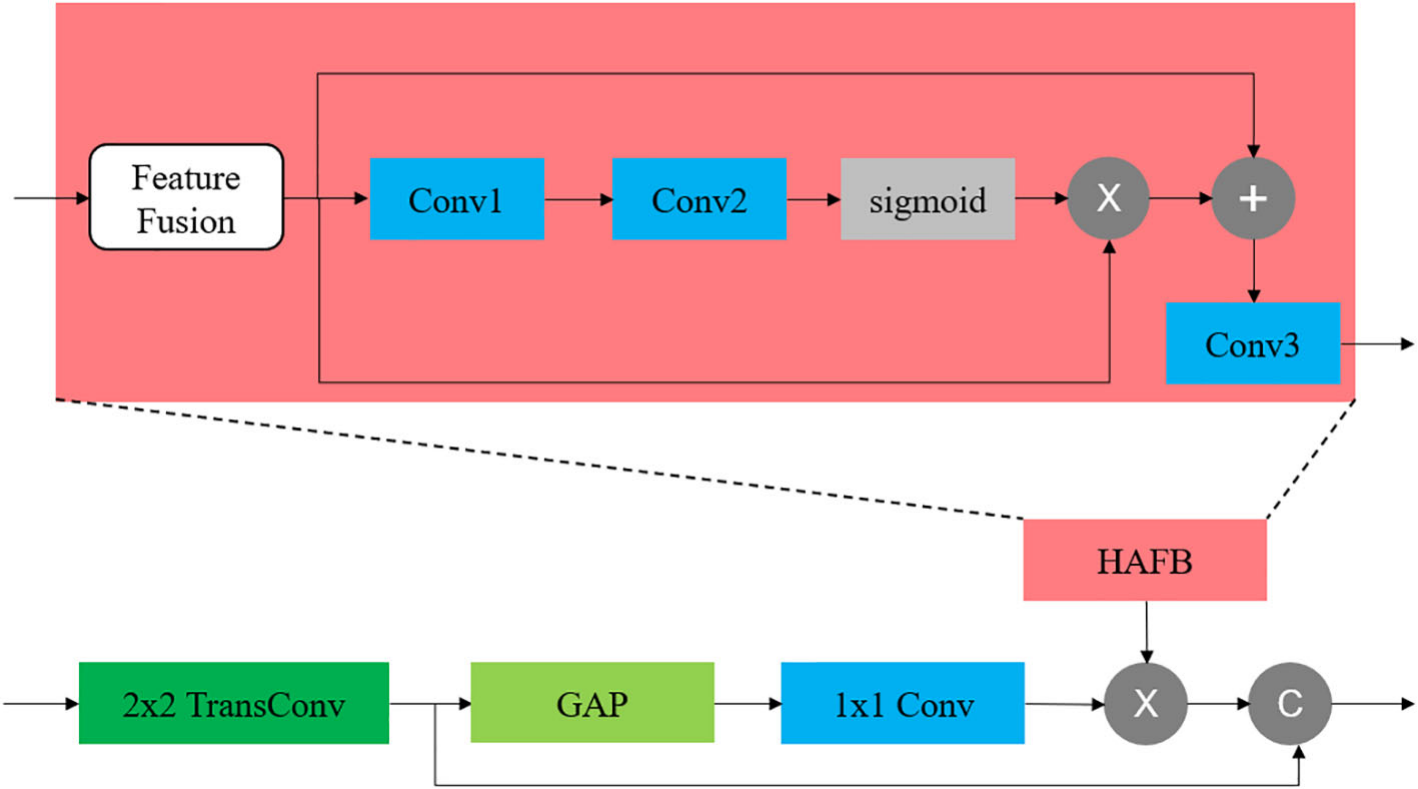
The location of HAFB in the network and its internal structure. The HAFB structure is introduced into the skip connection part. The feature fusion adopts the hybrid fusion method.


This fusion block can fuse multi-modal features from multiple encoder outputs in the skip connection stage, and filter more valuable features through the attention mechanism, which plays an important role in processing multi-modal images. In the network structure where HAFB is introduced, the skip connection stage does not just pass the results output by the encoder into the ecoder, but also needs to introduce an attention mechanism. The structure of HAFB is shown in **Figure 2**. The bottleneck layer and each layer of the encoder will output feature maps of multiple modalities. These feature maps are input into the corresponding HAFB, and these multi-modal feature maps are first fused into a single map, and then the useful information is selected through the attention mechanism. The fusion method used in this network is the fusion of the three strategies, including element summation, element product and element maximum value, and the channel-level splicing of the three feature maps can thus be obtained by,

(7) $$F=\left[\sum_{i=1}^{n}m_{i};\prod_{i=1}^{n}m_{i};\max\left\{m_{1},\ldots ,m_{n}\right\}\right]$$

where *n* is the number of modalities participating in the modal fusion and *F* means the feature map. This operation is applicable to any number of modalities. In this structure, the input multi-modal features are first fused into a feature map through the above-mentioned modal fusion strategy, and then the fused feature map is passed through an attention module,


(8) $$HAFB\;(F)=\varphi_{3}\left(F+F\times \sigma \left(\varphi_{2}\varphi_{1}F\right)\right)$$

where the convolution $\varphi_{1}$ is used to reduce the dimension of the feature map to $R^{C\times H\times W}$ , and then restored to $R^{3C\times H\times W}$ by the second layer of convolution $\varphi_{2}$ , thus to improve the expressive ability. In the above structure, the size of the convolution kernel of all convolutions used by the attention module is 3×3.

### 2.3 Self-supervised learning in multi-modal network

**Figure 3:**
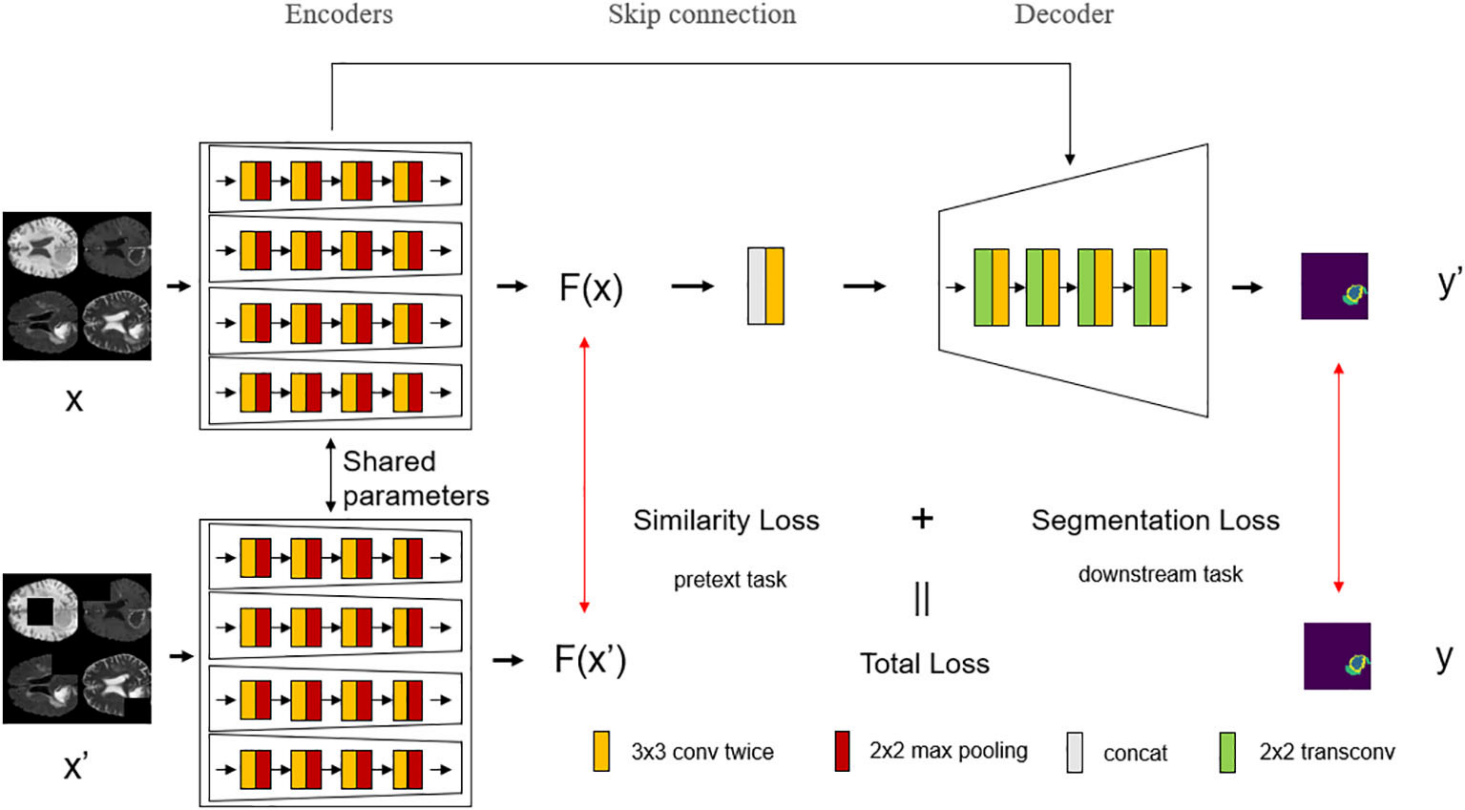
The overall structure of the model with the introduction of self-supervised learning. Self-supervised learning is introduced into the network, which divides the encoder stage into upper and lower branches, where the encoder shares parameters, and each modality of the multi-modal image input in the lower branch is randomly masked at different positions, and then trained according to the parameters of the shared encoder from the upper branch.

This work introduces a self-supervised learning mechanism based on the network structure, as shown in **Figure 3**. The encoder stage of this network structure consists of two branches, both of which use the multi-modal network structure described above as the backbone, and the encoder parameters of the two parts are the same, that is, the same encoder is used. In this work, in order to make full use of the complementary information between modalities, improve the robustness of the model, and make the network focus on the tumor region, masking is selected as the pretext task in the self-supervised learning, which can be seen as artificially adding noise to the image. When the down-branch encoder inputs an image, the image is first preprocessed, and an independent mask is taken for each input modality image. The method of masking the area preserves the connection between the modalities, and can realize the mutual complementation of the information between different modalities. It is worth noting that the positions of the occlusion regions of different modalities are different, otherwise the same occlusion position will not function as complementary information. The occlusion regions are different for different modalities to ensure that complementary information can be provided between modalities.

These masked multi-modal images are passed into multiple encoders of the lower branch as multiple inputs, and the same operations as the upper branch are performed. The upper branch has undergone the same operation, and it is only necessary to compare the similarity of the output results of the upper and lower branches to know whether the encoder in the network structure can make full use of the multi-modal complementary information for learning, and its robustness. The higher the similarity, the stronger the anti-noise ability of the encoder, and the better the use of multi-modal complementary information.

As shown in **Algorithm 1**, we present the training algorithm of the SM-ResUNet, which calculates the self-supervised labels required for network optimization, the prediction results of self-supervised tasks, and the prediction results of ownstream tasks during the training process.

## 3 Experiments

To verify the performance of the SM-ResUNet, we conduct visual analysis, ablation, and comparative experiments. Compared with the model without self-supervised learning and several classic models, the SM-ResUNet is generally better than them.

### 3.1 Dataset

The dataset used in this work is the multi-modal MRI brain tumor dataset BraTS 2019 and BraTS, 2020 (26, 27) Each patient has MRI images of four modalities, F1, F1CE, Flair, and T2. These MRI images are stereoscopic, showing the patient’s brain structure in each modality. Different values represent the lesion type at the corresponding location, and there are four labels, 0, 1, 2, and 4, respectively. Where 0 represents non-lesional area or background, 1 represents necrosis and non-enhancing tumor core, 2 represents peritumoral edema, and 4 represents enhancing tumor. Each image is of size 240×240×155 and needs to be sliced or trained using a 3D network during training. To make the input image size in the network structure suitable for the network, the middle 144 slices are taken and the image is cropped to 224×224×1.

Algorithm 1 Calculation of the Sm-Resunet during training. 
Input: image of size 
4×224×224
Initialization: 
modality←4,layer,ih[modality][layer]


x←
 tensor of image

$x^{'}\leftarrow$
 tensor of image with mask
//The process of Encoder
**for** 
t
 refers to 
$x,x^{'}$
 **do for** 
m←0
 to 
modality−1
 **do for** 
l←0
 to 
layer−1
 **do**

$t\left[m\right]\leftarrow ResConv_{E}\left[m\right]\left[l\right]\left(t\left[m\right]\right)$


ih[m][l]←t[m]


t[m]←MP(t[m])

**end for** 
t[m]←ASPP(t[m])
 **end for end for** 
$y\leftarrow ResConv_{N}\left(x\right)$
 //The process of Decoder
**for** 
l←layer−1
 to 0 **do**

y←TransConv[l](y)


y←[y;HAFB(ih[l])]


$y\leftarrow ResConv_{D}\left[l\right]\left(y\right)$
 **end for** 
y←EndConv(y)
 Output: 
$x,x^{'},y$

### 3.2 Implementation details

The CUDA version of the experimental platform is 11.4, and the graphics card model is NVIDIA GeForce RTX 3090. In this work, the initial number of convolution kernels is set to 32, and the batch size is set to 8. We use Adam as the optimizer and set the initial learning rate to 0.00001. The learning rate decays every 5 epochs with a decay rate of 0.9. A total of 15 epochs are set in this work, which can achieve the effect of loss value convergence.

The loss function used in this work is a mixture of dice loss and cross entropy loss. The overall loss function for the segmentation task is as follows,


(9) $$L_{seg}=\frac{\sum \left(\alpha L_{Dice}+\beta L_{CE}\right)}{M}$$

where α and β are set to 1 and 0.5. The advantage of using mixed loss is to prevent dice loss in some extreme cases, such as a very small proportion of a certain category of an image. The similarity loss is used to measure the similarity of the output results of the upper and lower branches in the self-supervised structure,


(10) $$L_{Similarity}=1-\frac{\sum \left(\theta (x)\times \theta (x')\right)+\epsilon }{\sqrt{\sum {\left(\theta (x)\right)}^{2}\times \sqrt{\sum {\left(\theta (x')\right)}^{2}+\epsilon }}}$$

where the smoothing coefficient ϵ=0.00001, θ(x) is the upper branch feature map and θ(x') is the lower branch feature map. The total loss function includes the loss from the self-supervised pretext task and the loss from the segmentation itself.

### 3.3 Evaluation metrics

On the official website that provides the BraTS dataset, the prediction results of the validation set can be evaluated. The evaluation indicators are derived from actual clinical applications and are divided into three categories: all tumor regions (WT), including all tumor structure regions; tumor core region (TC), including all tumor structures except edema regions; enhanced tumor regions (ET), which contains only one structure that enhances the tumor. For each category, there are several evaluation indicators used to calculate the score of the segmentation effect on the category, such as dice score, sensitivity, specificity and Hausdorff distance. Here, the distance in the 95th percentile of the length is used.

### 3.4 Experiment results

**Figure 4:**
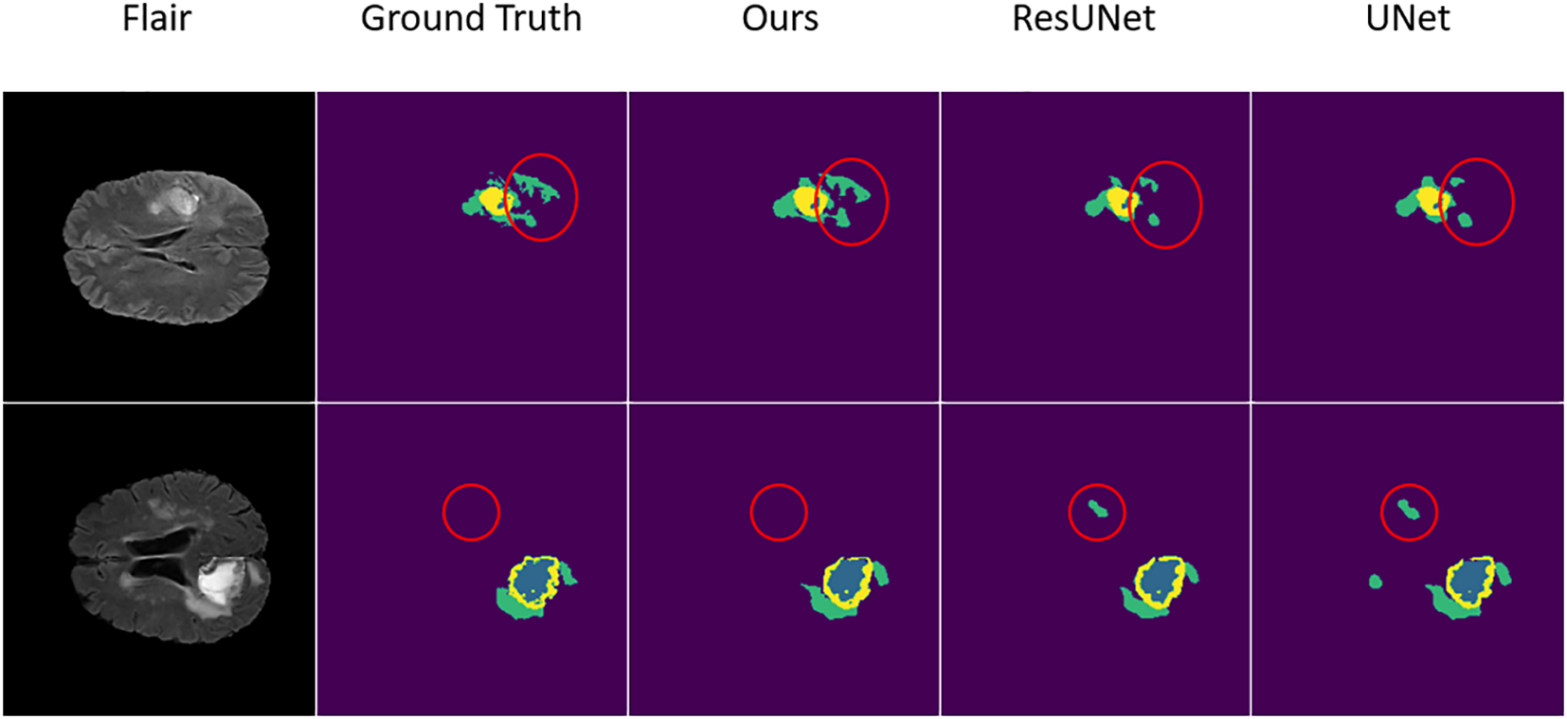
The result of the comparative experiment on BraTS 2019. As can be seen from the visualization of the segmentation results, after the comparative experiments, the SM-ResUNet is significantly better than other models, such as UNet and Res-UNet. The segmentation types in the figure are: non-tumor area or background (purple); necrotic and non-enhancing tumor core (blue); peritumoral edema (green); enhancing tumor (yellow).

In these prediction results, in order to better observe the performance, we randomly select several slices from the test set whose tumor area is not less than 5% of the entire image area, to prevent the tumor area from being too small to see the effect. From these visualization results, we can clearly feel the effectiveness of the network proposed in this paper for the brain tumor segmentation task. As shown in **Figure 4**, in regions with smaller brain tumors, the network is still able to accurately predict these regions.

After training the SM-ResUNet, in order to better evaluate the model, this work conducts comparative experiments with several state-of-the-art models, including the traditional single-modal U-Net, and the multi-modal models, namely, multi-modal Res-UNet, and multi-modal Res-UNet that introduces an attention mechanism module named Convolutional Block Block Attention Module (28). Since the single-modal U-Net itself does not have an advantage over other multi-modal models, it is set to 64 in the initial convolution kernel setting, which is twice as many as other models, and is trained for 10 more epochs. The comparison results are shown in **Table 1**. Through comparison, it is found that the effect of the SM-ResUNet is better than that of the compared models, which verifies that the model has better performance in medical segmentation tasks. On the BraTS 2019 dataset, although our model does not score better than some other innovative state-of-the-art methods in comparison (16, 18), our proposed approach that appropriately combines a multi-modal task with a self-supervised mechanism is of research value because such a self-supervised mechanism can assist the encoder in the complementary information between modalities fully exploit the complementary information between the modalities and further improve the anti-interference capability of the model as well as the ability to fill in the missing modalities. This approach may provide a new design idea for future self-supervised multi-modal medical image segmentation, making full use of multi-modal-specific information for self-supervised training, rather than simply superimposing the two training methods.

**Table 1:** Comparative experimental results on BraTS 2019.

Methods	Dice (%)		
	ET	WT	TC
U-Net (6)	69.679	85.733	73.364
Multi-modal Res-UNet	67.596	80.666	71.561
Multi-modal Res-UNet + CBAM (28)	69.975	85.237	69.573
Ours	**70.689**	**86.268**	**73.998**

The bold values are the best result.

**Figure 5:**
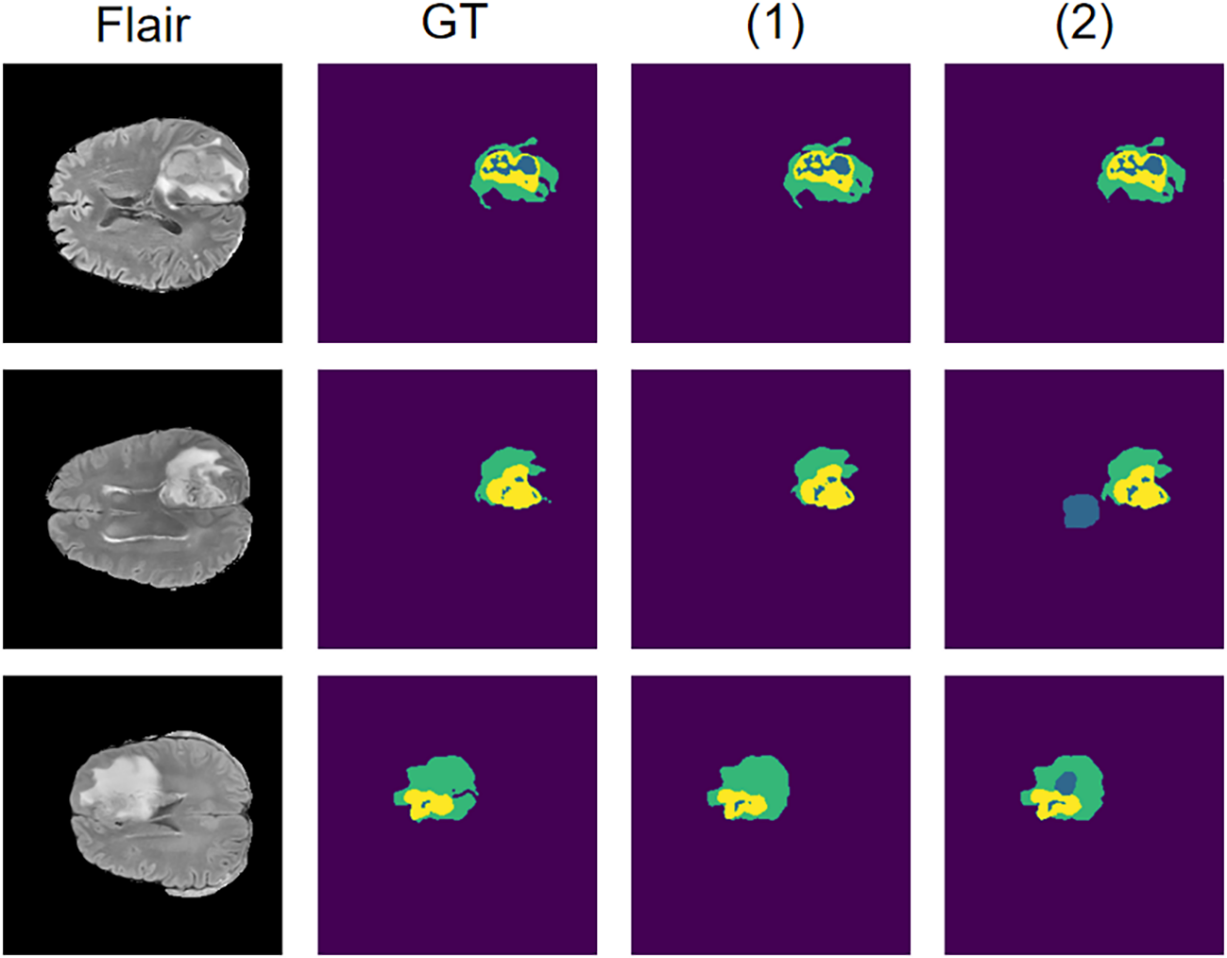
Results on the BraTS 2020 dataset using the self-supervised network compared to the network without self-supervision. Where (1) represents the network where the self-supervised training is introduced and (2) represents the network without self-supervised training.

We compare our model with the state-of-the-art models on the BraTS 2020 validation set, with experimental data from Li et al. (32). The experimental data shows that our model obtains the best results on ET and WT (**Table 2**). Although the networks are able to achieve better scores on the BraTS 2020 dataset both before and after the addition of self-supervision, the self-supervised network is still able to outperform. We analyze that this may be due to the fact that the network is able to extract more information in the encoder with self-supervision, which allows the encoder to handle the detail part better, as shown in **Figure 5**, and thus achieve a higher score.

**Table 2:** Comparative experimental results on BraTS 2020.

Methods	Dice (%)			Hausdorff 95			FLOPs(G)
	ET	WT	TC	ET	WT	TC	
3D U-Net (29)	68.76	84.11	**79.06**	50.983	13.366	13.607	1,669.53
Basic V-Net (30)	61.79	84.63	75.26	47.702	20.407	**12.175**	749.29
Deeper V-Net (30)	68.97	86.11	77.9	43.518	14.499	16.153	–
3D Residual U-Net (31)	71.63	82.46	76.47	37.422	12.337	13.105	407.37
Ours	**73.49**	**86.84**	74.12	**25.537**	**6.416**	23.439	**344.08**

The bold values are the best result.

In addition, to validate the effectiveness of this self-supervised strategy on a small amount of data, we conduct separate comparison experiments on the BraTS 2020 dataset with and without self-supervised learning approach. Both experiments use only 20% of the training data. The dice coefficients of the model with self-supervised learning are 65.074%, 78.352% and 61.858% on ET, WT and TC, compared to 58.629%, 65.535% and 55.966% for the model without self-supervised learning. Although there is a significant decrease in model accuracy after using a small amount of data, all evaluation metrics are significantly higher on the model with the addition of self-supervised learning than on the model without self-supervised learning, and still achieve more accurate segmentation. This indicates that this self-supervised strategy can still be beneficial for training on a small amount of data.

### 3.5 Visualization of the HAFB

**Figure 6:**
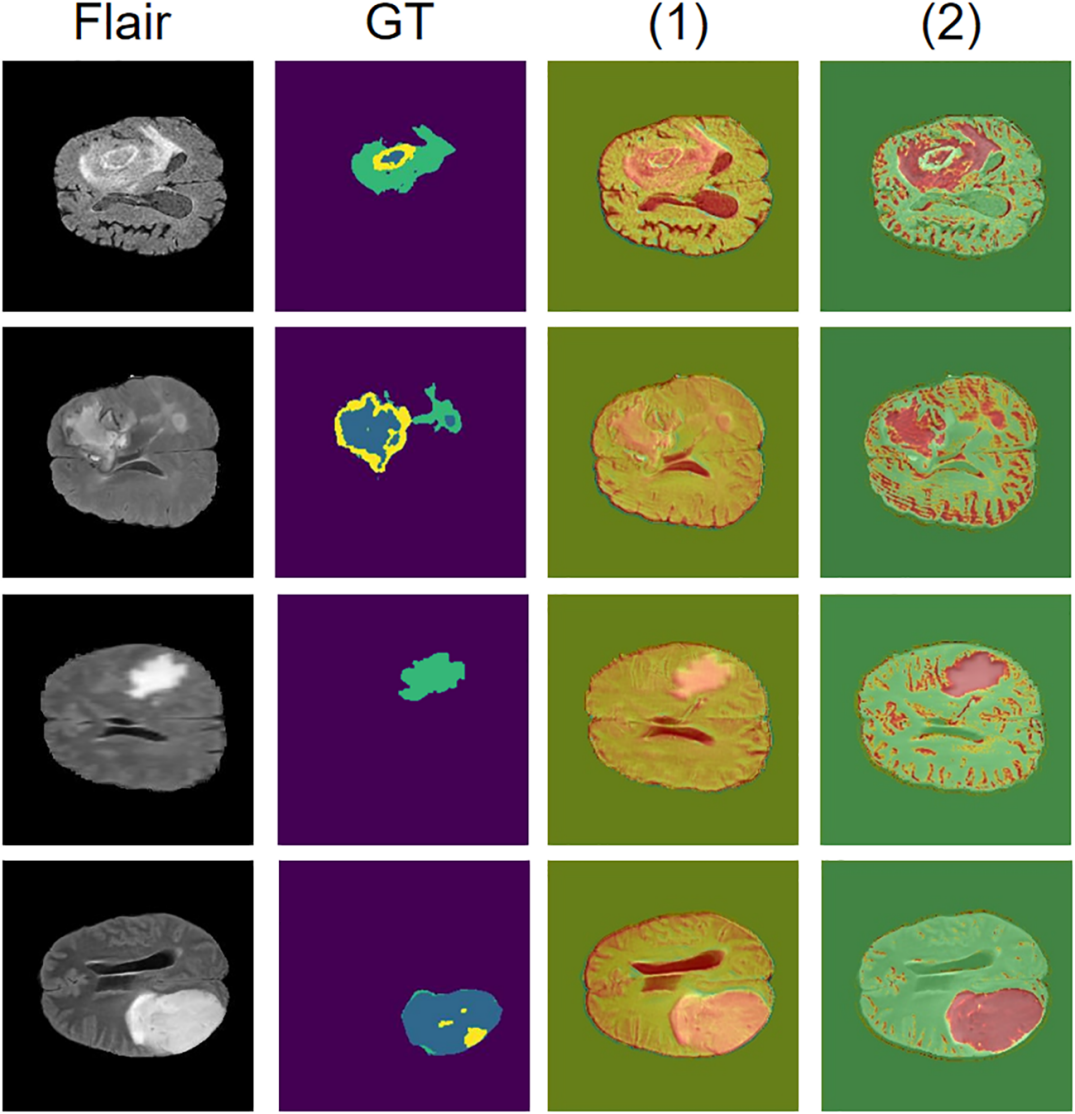
Visualization of attention mechanisms. The Flair modality is used as the original image, GT is the ground truth of the segmentation result, and the red area is the region of interest to the network, where (1) is the network model without HAFB and (2) is the network model with HAFB introduced.

To show the performance of the attention mechanism in our HAFB module and its effectiveness, we visualize and analyze the attention feature maps computed in it. In this structure, the attention feature map is computed after the modal fusion and the attention is paid to the feature map after the modal fusion. We demonstrate here the effect of the attention mechanism in the HAFB module using the Flair modality as an example, as shown in **Figure 6**. The red region is the high scoring region, which indicates the network has higher interest in this part of the region. From the visualization results, the HAFB module can generate higher interest to the tumor region in the feature map after modal fusion, providing segmentation focus for the later network structure, and improving the accuracy of the whole network. Since the network model without the HAFB module does not have the calculation of attention at the skip connection, we use the activation map (33) corresponding to the feature map there to show the region of interest in the network. Although the model without the addition of HAFB also pays more or less attention to the tumor region, the effect is not significant compared to the model with the addition of HAFB.

### 3.6 Ablation study

The self-supervised learning mechanism is used in the process of training the SM-ResUNet. In the ablation experiment, the segmentation results of Res-UNet with a self-supervised learning mechanism and Res-UNet without a self-supervised learning mechanism are compared to prove the necessity of introducing a self-supervised learning mechanism into the network structure. As can be seen from **Table 3**, after the introduction of self-supervised learning, most of the indicators have been improved by different degrees. In order to facilitate the comparison, the scores of different indicators of the three types of tumor regions are averaged for comparison. We have bolded the data with better results in the two groups of comparisons. It is not difficult to see that after the introduction of the self-supervised training mechanism, the network can basically achieve meaningful tumor region segmentation accuracy in practical clinical applications.

**Table 3:** Ablation experiment results on BraTS 2019.

Methods	Supervision	Dice (%)	Sensitivity (%)	Hausdorff 95
Multi-modal UNet+ASPP+HAFB	Fully supervised	76.604	77.081	**9.29248**
	Self-supervised	**77.185**	**78.375**	12.0272
Multi-modal Res-UNet+ASPP+HAFB	Fully supervised	76.070	76.536	8.69363
	Self-supervised	**76.985**	**79.308**	**8.19243**

The bold values are the best result.

We perform the analysis of the ablation experiments for the ASPP and HAFB modules, as shown in **Table 4**, which are performed using the self-supervised strategy. The experiments show that with the ASPP module, although there is no significant improvement in the dice coefficient, there is a more significant improvement on Hausdorff 95. We speculate that this is because the ASPP module performs feature extraction from multiple scales on the feature map at the end of the encoder, which makes it more accurate for edge information extraction. The improvement of the multi-modal model with the addition of the HAFB module is more significant, in terms of the dice coefficient and Hausdorff 95. To verify its effect on single-modal model, we conduct ablation experiments on the HAFB module with the single-modal model and find that its effect does not work as well as the multi-modal model. This may be due to the fact that the module loses some information in the feature maps during the computation, and thus is not as good as using the original feature maps directly on the single-modal model. However, it is beneficial to extract valuable information on each modality feature map on multi-modal models, thus avoiding redundancy and improving the model’s ability to extract multi-modal features.

**Table 4:** Ablation experiment results on BraTS 2020.

Methods	Dice (%)			Hausdorff 95		
	ET	WT	TC	ET	WT	TC
Res-UNet+ASPP	69.911	85.987	74.176	35.823	6.501	19.871
Res-UNet+ASPP+HAFB	71.728	85.125	66.699	31.912	9.521	38.239
Multi-modal Res-UNet+ASPP	70.745	85.432	69.815	32.317	9.015	30.47
Multi-modal Res-UNet+HAFB	72.652	87.012	76.084	32.214	7.819	22.282
Multi-modal Res-UNet+ASPP+HAFB	73.487	86.838	74.124	25.537	6.416	23.439

To investigate the effect of the HAFB module on the network scale, we test it in single-modal without HAFB, single-modal with HAFB, multi-modal without HAFB, and multi-modal with HAFB networks using data with 1 to 6 modalities, and record the results as shown in **Table 5**. It can be seen that the HAFB module in singe-modal networks cannot play a role in reducing the network scale, which is as expected, because in the single-modal networks there is no need to fuse the feature maps of multiple modalities, but to calculate them as a whole in the network, and the HAFB module will expand its channel count up to three times. In a multi-modal network, the more the number of modalities of the data, the more the number of parameters the HAFB module can reduce. At three modalities the two are similar, with each additional modality not using the HAFB module increasing the number of parameters much more than the network with the HAFB module. This is because the HAFB module is able to stabilize the number of channels after modal fusion at three times the previous number (because of the fusion of each modality feature from three aspects), while the number of channels of the decoder without the HAFB module will be several times the number of modalities of the encoder.

**Table 5:** Comparison of the number of model parameters (M).

Number of Modalities	Single-modal	Single-modal+HAFB	Multi-modal	Multi-modal+HAFB
1	16.95	45.255	16.95	45.255
2	16.951	45.255	40.167	59.067
3	16.951	45.256	69.651	72.879
4	16.951	45.256	105.402	86.69
5	16.952	45.256	147.42	100.502
6	16.952	45.257	195.705	114.314

**Figure 7:**
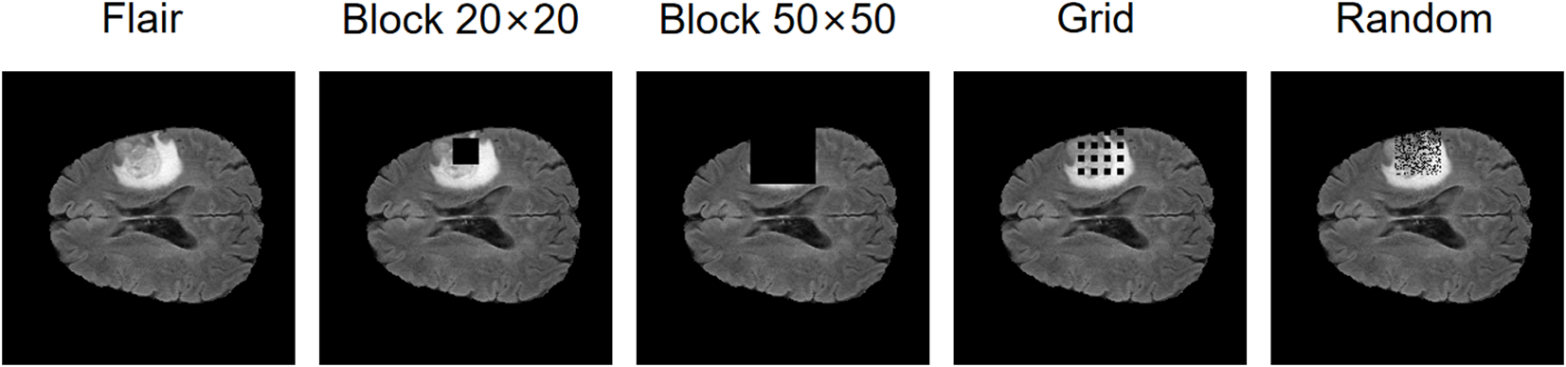
The effect of masking strategies that we design.

In addition, we conduct an ablation study on the masking strategies, shown in **Table 6**. In each experiment, the control variables method is used to study the mask area and dispersion degree respectively. Specifically, the original mask form is a square of size 20×20, and we design three masking strategies separately: changing it to 50×50 size with the same form; the form is changed to a grid-like distribution with 4×4 = 16 square masks of size 5×5 uniformly distributed within the range of 35×35 pixels, each mask spaces by 5 pixel points, whose total area is the same as that of the 20×20 size mask; the form is changed to a random distribution with 400 pixel points randomly masked off within 35×35 pixels (keeping the same as the second masking strategy), and its total area is the same as that of the 20×20 size mask. These three masking strategies are shown in **Figure 7**. The experiments show that all these masking strategies are able to make the self-supervised strategy effective and make the model accuracy improve. Overall, the best performer is the 20×20 square mask, which achieves the highest segmentation accuracy on ET in terms of dice coefficient and Hausdorff 95, while the grid-like as well as random masks has a slightly higher dice coefficient on WT and TC than the other strategies, which may be due to its large range and ability to cover more information, improving the training effect of the self-supervised strategy.

**Table 6:** Ablation experiment results on the masking strategies on BraTS 2020.

Methods	Dice (%)			Hausdorff 95		
	ET	WT	TC	ET	WT	TC
Block 20×20	**73.487**	86.838	74.124	**25.537**	**6.416**	23.439
Block 50×50	72.334	86.585	73.786	28.225	6.463	**15.603**
Grid	72.755	**87.36**	74.478	28.593	8.122	22.061
Random	72.954	85.49	**75.228**	30.75	6.474	18.757

The bold values are the best result.

## 4 Conclusion

In this paper, we propose the SM-ResUNet, which can learn the independent features from different modalities. We enable the network to learn multi-modal features by introducing multiple encoders, and employ a self-supervised learning approach to fully utilize the dataset for training. Moreover, a pretext task in self-supervised learning is explored to assist the SM-ResUNet in training and improve the robustness of the network. Thus, it can not only retain the information corresponding to the original multi-modal image, but also enabling the network to learn the complementary information between the modalities. In addition, the HAFB module is integrated to the network to extract the features of multiple modalities, and fix the number of channels of the feature map favorably, so that the network can be fixed to a stable structure. Experiments on BraTS show that the SM-ResUNet is superior to the compared methods. This is because the SM-ResUNet can learn complementary information from multiple modalities, and alleviate the problem of high noise in medical images to a certain extent.

## Data availability statement

Publicly available datasets were analyzed in this study. This data can be found here: BraTS 2019, BraTS 2020.

## Author contributions

LZ contributes to the idea; CJ contributes to the method and writing; YS and JM contribute to the experiments; ZL contributes to the data; HY contributes to the validation. All authors contributed to the article and approved the submitted version.
